# Supplementary material for: Murine leukemia virus glycoGag antagonizes SERINC5 via ER-phagy receptor RETREG1
Source: PLoS Pathog. 2025 Oct 9;21(10):e1013023. doi: 10.1371/journal.ppat.1013023 (PMC12530543; doi:10.1371/journal.ppat.1013023)
Supplement: S2 Fig — (PDF) [file ppat.1013023.s002.pdf]

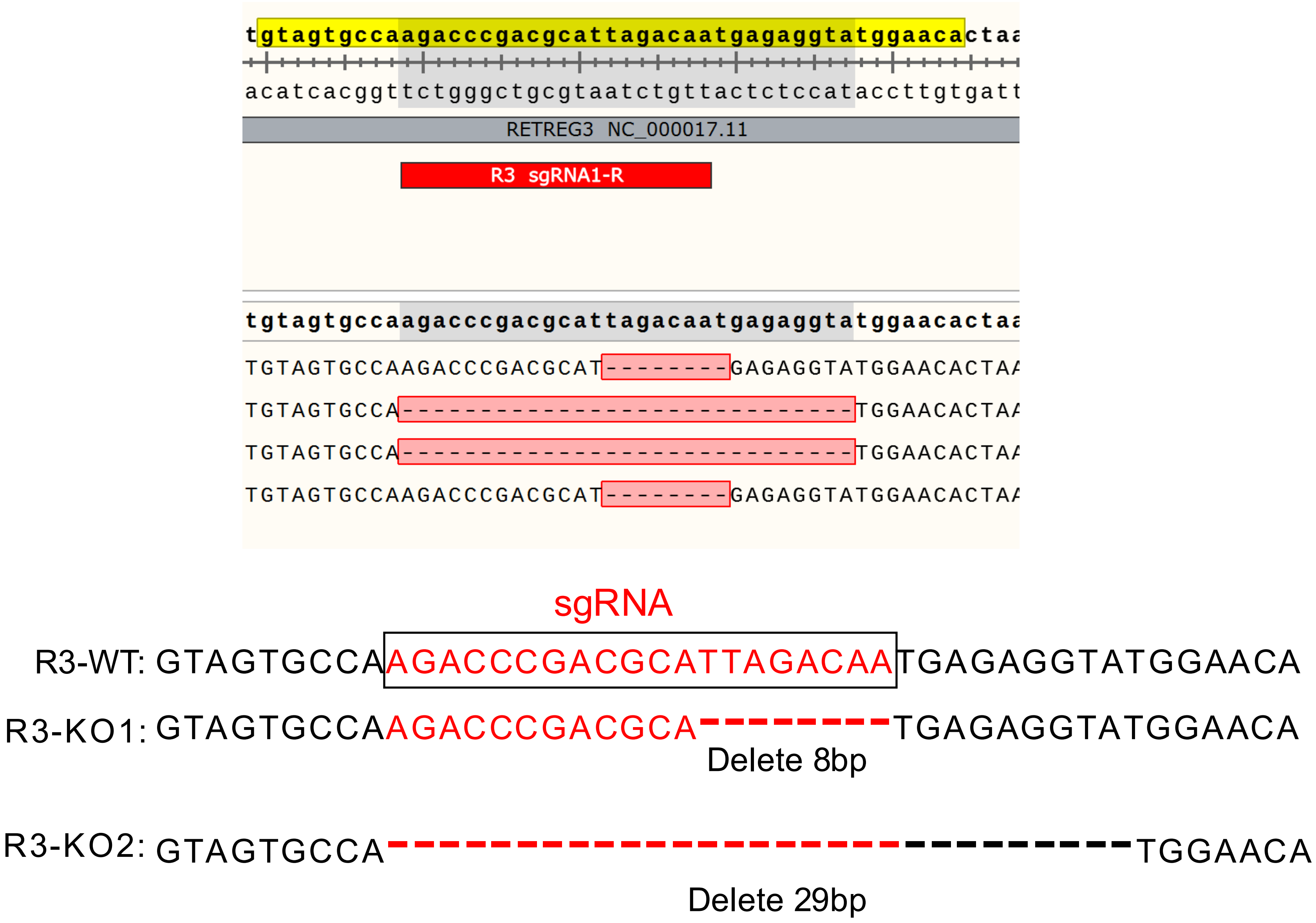

S2_Fig. Validation of HEK293T *RETREG3 (R3)*-KO cells by genomic sequencing.
