# Supplementary figures and images for: Murine leukemia virus glycoGag antagonizes SERINC5 via ER-phagy receptor RETREG1
Source: PLoS Pathog. 2025 Oct 9;21(10):e1013023. doi: 10.1371/journal.ppat.1013023 (PMC12530543; doi:10.1371/journal.ppat.1013023)

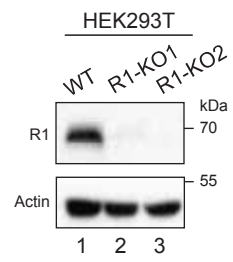

**S4\_Fig.** Validation of *R1*-KO clones by WB.

Supplement: S4 Fig — (PDF) [file ppat.1013023.s004.pdf]
