# Supplementary material for: Murine leukemia virus glycoGag antagonizes SERINC5 via ER-phagy receptor RETREG1
Source: PLoS Pathog. 2025 Oct 9;21(10):e1013023. doi: 10.1371/journal.ppat.1013023 (PMC12530543; doi:10.1371/journal.ppat.1013023)
Supplement: S5 Fig — RETREG2 was expressed with its specific siRNAs (#1, #2) or a control (Ctrl) in HEK293T cells and its expression was detected by WB. (PDF) [file ppat.1013023.s005.pdf]

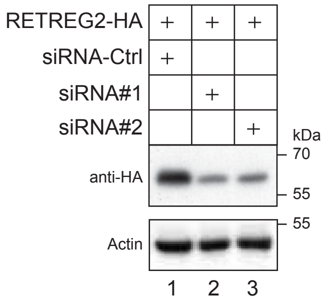


**S5_Fig.** Validation of *RETREG2* siRNAs. RETREG2 was expressed with its specific siRNAs (#1, #2) or a control (Ctrl) in HEK293T cells and its expression was detected by WB.
