# Supplementary material for: Murine leukemia virus glycoGag antagonizes SERINC5 via ER-phagy receptor RETREG1
Source: PLoS Pathog. 2025 Oct 9;21(10):e1013023. doi: 10.1371/journal.ppat.1013023 (PMC12530543; doi:10.1371/journal.ppat.1013023)
Supplement: S6 Fig — Ser5 and K130R were expressed with glycoMA in HEK293T cells in the presence of LAMP2a-specific siRNA or its control (Ctrl). Protein expression was detected by WB. (PDF) [file ppat.1013023.s006.pdf]

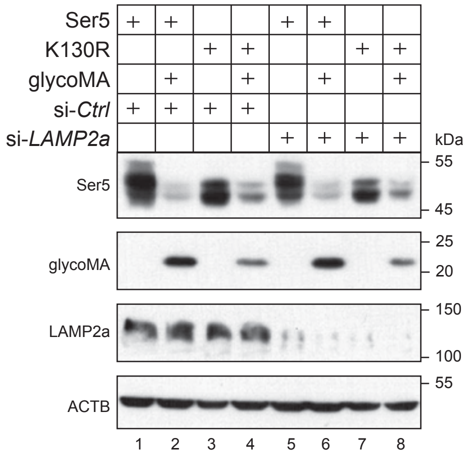

S6_Fig. LAMP2a is not required for glycoMA downregulation of Ser5. Ser5 and K130R were expressed with glycoMA in HEK293T cells in the presence of *LAMP2a*-specific siRNA or its control (Ctrl). Protein expression was detected by WB.
