# Supplementary material for: Murine leukemia virus glycoGag antagonizes SERINC5 via ER-phagy receptor RETREG1
Source: PLoS Pathog. 2025 Oct 9;21(10):e1013023. doi: 10.1371/journal.ppat.1013023 (PMC12530543; doi:10.1371/journal.ppat.1013023)
Supplement: Attachment — Submitted filename: Point-by-point response .docx [file ppat.1013023.s008.docx]

**Summary:**

We have addressed the concerns and included new results. These are the major changes:

1. **New Results**: Presented in Fig.4G, Fig.7D, Fig.7E, Fig.S1, Fig.S2, Fig.S3, and Fig.S4.
2. **Updated Images**: Fig.3A fluorescence image is updated.
3. **Quantifications**: Provided in Fig.5B, Fig.6C, Fig.6E, Fig.7A, Fig.7C, Fig.7D, and Fig.8B.
4. **Statistics**: Detailed in all quantitative figures, and the number of repeats is provided in the figure legends.

Due to these changes, three new authors are included. We thank the reviewers for their constructive comments, which have helped improve this manuscript significantly.

**Reviewer #1:** Ahmad et al. report a mechanism enabling the retroviral accessory protein glycoGag to counteract the restriction factor SERINC5. Specifically, the authors identify that glycoGag uses a strategy to antagonize SERINC5 that is distinct from how Nef antagonizes SERINC5. The authors report that glycoGag interacts with SERINC5 in the endoplasmic reticulum and triggers SERINC5 degradation through ER-phagy. This conclusion is supported by AP-MS data showing interaction between glycoGag and ER-phagy receptors such as RETREG1. The authors also show that glycoGag loses the capacity to antagonize SERINC5 in cells deficient for RETREG1. Additionally, the authors report some interesting results regarding how Nef downmodulates SERINC5. It appears to do so in a manner that requires p62 and PI3KC3, which are proteins normally associated with macroautophagy. This study is well-written and nicely organized and presented, with carefully crafted figures. However, I believe there are a number of changes that could be introduced to improve the manuscript.

1. In Figure 1B, Nef only minimally restores infectivity of DeltaNef HIV-1 infectivity. Authors should comment on this result. Furthermore, in Figure 1C, the authors should address levels of SERINC5 in virions, not just producer cell lysates. Many papers studying Nef/glycoGag have not observed a decrease in SERINC5 levels in producer cells. I think Figure 1D should be removed, since it is an arbitrary, author-defined definition of “antagonism” that takes into account detected Nef and glycoMA protein. It is unnecessary to make a figure to make this point.

Figure 1D has been removed.

2. When addressing glycoGag stability and activity, including where it localizes to and how it is degraded, the degree to which glycoGag is expressed is very important. Previous papers (PMID: 27879338 and PMID: 31964738) have shown that only a very small amount of glycoGag is required to counteract SERINC5/IFITM3 and rescue MLV infectivity. Conversely, transfection of elevated levels of glycoGag plasmid causes loss of virus infectivity for unknown reasons. This could be discussed in the final paragraph of the Discussion, when authors speculate about why glycoGag is rapidly turned over in proteasomes.

The stability of glycoGag was previously discussed in the final paragraph of an earlier submission to another journal. In the current version, this discussion has been incorporated into the second paragraph.

We have cited these two references to support the discussion and help clarify the underlying mechanism.

3. I disagree with the author’s interpretation of Figure 4I and 4J. The results show that CCNK knockdown and CDK13 shRNA are important for the downmodulation of Ser5 by glycoMA. The effect is partially but lanes 6 and 8 should be compared. There is a definite rescue of Ser5 protein by CCNK shRNA and CDK13 shRNA. Furthermore, the authors have duplicated a membrane in Figure 4I and 4J. On the right side, the Ser5 blots are identical in 4I and 4J. Even though the brightness/contrast of the two images is different, it is clearly the same membrane. This should be corrected through independent experiments.

We thank this reviewer for carefully reading our figures. However, we confirm that the Ser5 blots in Figures 4I and 4J (now, merged into new Figure 4I) are not from the same membrane. Please compare the original images for these two figures:

Regarding the partial disruption of the glycoGag activity in *CCNK* and *CDK13* knockdown experiments, we have revised the text to state these results more accurately.

4. The authors base a number of their conclusions on experiments utilizing 3-MA and Ly, which are non-specific inhibitors of PI3K family members as well as mTOR. The authors should include an experiment with SAR405, a specific inhibitor of PI3KC3 (vps34) and which inhibits production of PI3P, to confirm that macroautophagy is not responsible for turning over SERINC5 in glycoGag expressing cells. A paper that has successfully used SAR405 to dismiss a role for macroautophagy is PMID: 36264642.

We have already knocked out *PI3KC3* (*Vps34*), but did not detect any effect (Fig.8A, lanes 71, 72). It is unnecessary repeat this experiment with this inhibitor.

5. The full dataset of the AP-MS needs to be provided in the supplemental information. The reviewers and readers should be able to see the full set of proteins shown to interact with Nef and glycoGag, as well as their relative abundances.

The full dataset was already deposited to iProX before this submission, which can be downloaded (project ID: IPX0010260000, see Methods, Mass Spectrometry section).

6. In Figure 3E, why is SERINC5 K130R cotransfected with RETREG1 (left part of figure) while SERINC5 WT is cotransfected with RETREG1-2 isoform? I think that SERINC5 WT should feature throughout Figure 3E.

This reviewer is referring to a previous submission of this manuscript to another journal. The current version does not include the outdated figure mentioned. We have already incorporated SERINC5 WT, as seen in Figure 7D.

7. I do not think that the authors do a convincing job of distinguishing between macro-ER-phagy and micro-ER-phagy. The authors claim in the Results and Discussion sections that their results with glycoGag suggest that it is using the micro-ER-phagy pathway to degrade SERINC5 WT and K130R, but they do not point to the specific data result that led them to that conclusion. I would suppose that they come to this conclusion because ATG5 and ATG7 are not required for glycoGag-mediated degradation of SERINC5. Since ATG5 and ATG7 are important for autophagosome function during macroautophagy, but it is not clear to me whether ATG5 and ATG7 are required for macro-ER-phagy. I would argue that it is not important for them to make the distinction between macro-ER-phagy and micro-ER-phagy when interpreting their results. It is sufficient to indicate that ER-phagy mediated by RETREG1 is the mechanism by which glycoGag antagonizes SERINC5.

Macroautophagy depends on autophagosome biosynthesis machinery, including PIK3C3/VPS34 and Beclin 1 (BECN1), as well as the MAP1LC3/LC3 lipidation machinery involving ATG3, ATG5, and ATG7. We knocked out these genes and found that glycoGag still antagonizes SERINC5. Therefore, macroautophagy is unlikely to be involved in this antagonism.

8. It is possible that, if the authors studied various isolates of Nef and glycoGag, their findings could be different. To what extent is the use of ER-phagy by glycoGag conserved, for example? Perhaps some isolates of Nef also use ER-phagy to antagonize Ser5?

This is an interesting question, which will be addressed in our future studies.

Minor:
1. If authors intend to state that glycoGag and glycoMA exhibit an intracellular distribution, as opposed to localization to the plasma membrane, they should not use the term “cytoplasmic localization” to describe the location of glycoGag and glycoMA. As they show, these proteins colocalize with the ER marker CALR. As transmembrane proteins, they are membrane bound and therefore not technically “cytoplasmic.” It is better to remove that descriptor and indicate that they are found intracellularly in ER membranes.

“Cytoplasmic” refers to all components within the cell membrane, including organelles, the cytoskeleton, and the cytosol. In contrast, “cytosol” specifically denotes the fluid portion of the cytoplasm, excluding organelles and other membrane-bound structures. Therefore, our description is accurate.

2. “Transiently knocked down using CRISPR/Cas9” should be replaced with “knocked out using CRISPR/Cas9 in a population of cells.”

The phrase 'in a population of cells' is unclear, and we do not know how to address this concern.

3. In Figure 3, HEK293T are mislabeled as “HEK393T.”

This reviewer is still referring to a figure that was included in a previous version of the manuscript but has since been removed.

4. Can the authors comment on why they think SERINC5 protein levels increase in p62 KO and PI3KC3 KO cells? Also, why does SERINC5 protein level decrease upon ATG3 KO or ATG5 KO? These findings are unexpected and interesting.

We speculate that SQSTM1/p62 is involved in protein degradation, whereas ATG3 and ATG5 are important for cell growth.

5. Typo in Figure 6 (“glucoGag”)

It has been corrected.

6. Typo in Figure 8 (“knoctouts”)

It has been corrected.

**Reviewer #2:** In this manuscript, Ahmad and co-authors provide details on the molecular mechanism by which glycoGag downregulates SERINC5. Strong evidence shows that the mechanism is different between Nef (the Serinc5 antagonist in HIV-1) and glycoGag (the antagonist in MLV) with data supporting a role for ER-phagy as the preferred method by which glycoGag accomplishes this. The manuscript is well-written, and the data presented is of high quality. However, the authors at times make overstatements from their data, which should be accompanied by additional experiments to unequivocally support their conclusions.

Fig. 1. Experiments should be reproduced in cells expressing full-length glycoGag.

The active domain of glycoGag was mapped to glycoMA by Pizzato (2010, PNAS) and later confirmed by Gottlinger (2014, JVI). Both groups also demonstrated that glycoGag and glycoMA are functionally interchangeable in counteracting SERINC5 (2015, two Nature papers). Our study shows that these proteins share identical biochemical properties (Fig. 2A, 2B, 2D, and Fig. 3A). Given this redundancy, repeating these experiments in Figure 1 is unnecessary.

Fig. 3B. No controls to show purity of the membrane fractions are included. Additionally, making conclusions on how SER5 affects the subcellular distribution of glycoMA by looking at subcellular fractionation assays is an overstatement. The authors should assess this by fluorescence microscopy and accompany the images with quantifications.

We confirmed the membrane purity by showing that the membrane fractions do not contain actin in Fig.3B. In addition, the purity of our membrane fractionation can be confirmed from Fig.4C, where we show that both K130R and K131R mutants were not detected from the membrane fraction (see below).

We used Western blotting to show that SER5 could recruit glycoMA to the plasma membrane, since this redistribution could not be detected by fluorescence microscopy due to the lower sensitivity of this method compared to Western blotting.

Fig. 4 F/H/I. The conclusions derived from these panels (that glycoMA downregulates SER5 from both the cell surface and cytoplasm) are an overstatement. The blots presented represent to whole cell lysates (not fractionations). Moreover, the results in panel G, where most SER5 appears in intracellular compartments in the presence of glycoMA, may represent an intermediate phenotype of glycoMA-dependent downregulation of SER5. To drive conclusions on whether glycoMA can downregulate SER5 present at intracellular membranes, authors should run experiments in the presence of inhibitors of ER to membrane transport or a similar approach.

We previously reported that the SERINC5 K130R mutant—unable to be polyubiquitinated by Cul3-KLHL20—is displaced from the plasma membrane (*Nat Commun*, 2022; PMID: 35474067). In this study, we further confirmed that K130R is not expressed on the plasma membrane (Fig. 4C). Therefore, we refer to the K130R mutant as cytoplasmic SERINC5 in Fig. 4F/H/I, without the need for additional fractionation.

Unlike other approaches, such as blocking protein trafficking from the ER to the Golgi, this well-characterized SERINC5 mutant provides direct evidence that glycoGag can downregulate SERINC5 even when it is not present on the cell surface.

Fig. 6B. Claims about RETREG1’s role in the downregulation of SER5 by glycoGag should be accompanied by quantifications from at least 3 independent experiments.
Statistics: no statistics are provided. Most of the data is derived from duplicate experiments.

As suggested by this reviewer, the quantifications from 3 independent expressions are now provided in Fig.6C. In addition, statistics and numbers of repeats are provided in all figure legends.

Line 90 change “downregulates” by “downregulate”

It has been corrected.

Fig. 2. To confirm specificity, the authors should silence another E3 ligase complex.

The interaction of Cul3-KLHL20 with glycoGag was also reported elsewhere (PMID: 37787515).

Fig. 3. Besides localizing at the plasma membrane, Nef is also present in the cytoplasm (also confirmed in their Fig. 2B data). The selection of images in Fig. 3A does not allow to see Nef’s cytoplasmic localization, probably because the cells selected are close to each other and the cytosol is much smaller than the panels for glycol-Gag and glyco-MA. Authors should select for cells with similar characteristics.

We have adjusted the background for the Nef expression. The cytoplasmic Nef is now visible in the new image (Fig.3A).

Fig. 3B. GlycoMA is detected also in the membrane fraction in cells expressing SER5. This needs to be acknowledged in the results section (lines 141-142). Also, add the ladder marker for the Nef and glycoMA membrane, as it is difficult to discriminate whether the bands correspond to Nef or glycoMA.
This result had been described in line 147 of this initial submission. Markers have been included to indicate the sizes for Nef and glycoMA in Fig.3B.

Fig. 4E. The authors should clarify that here both Nef and glycoMA have the same tag. Also, molecular weight indicators are encouraged throughout the manuscript.
As suggested by this reviewer, this information is now included in the legend.

Lines 145-146. None of the conditions in Fig. 3B have Nef and glycoMA expressed together. So, I am unsure what the authors are trying to claim when they say with the statement “when co-expressed with Nef and glycoMA, Ser5 did not alter the levels of Nef in the cytosolic and membrane fractions”.
In this sentence, “and” has been replaced with “or”.

Lines 263-264. The conclusion that glycoGag downregulates SER5 via LC3-dependent transport or CMA is an overstatement here. The authors should change the language saying that the findings in Fig 8A suggest that glycoGag downregulates SER5 via micro-ER-phagy. To make such conclusion, the authors should use drugs specific for micro-ER-phagy (3-MA, Baf-A1 nor Ly target this type of autophagy in a specific manner), or silence genes that are specific for this pathway other than RETREG-1.

We have already used 3-MA and Baf-A1 (Fig.5A, Fig.8B) and obtained consistent results. We have also knocked out or silenced the major genes in macro-ER-phagy and CMA. It is unclear which specific gene for micro-ER-phagy should be further investigated.

Fig 5B. Staining for Nef and glycoMA is missing. Without detecting these proteins in the selected images, we cannot know if the cells selected were indeed Nef+ or glycoMA+.

Our transfection efficiency ranges from 60-80% in these experiments. We have demonstrated the expression of Nef and glycoMA using Western blotting. Moreover, by including the empty vector as a control, we believe the images were not derived from Nef- or glycoMA-negative cells.

Fig. 6. Each panel is lacking the labeling in one of the membranes (jusy above the actin membrane). I am assuming this corresponds to each ER-phagy receptor being tested, but this needs to be labeled.

The labels are added in parallel in this figure, as suggested.

Fig. 7E. Without adding a beads-only and an IgG-only control, the authors cannot conclude that RETREG1 is only present in the pulldown fraction of cells co-expressing SER5 and glycoGag, as there are bands with similar molecular weight in the SER5 only and SER5 + Nef samples.

This control is now included as a new result in Fig.7E, lane 16.

Fig. 8. The authors need to provide evidence showing lack of expression of target genes in the knockout cells.

We utilized our previously reported HEK293T knockout cell lines for *ATG3*, *ATG5*, *ATG7*, *BECN1*, and *PIK3C3* (Ref. 26), as well as *CCPG1*, *RTN3L*, *ATL3*, and *SEC62* (Ref. 42). In this study, we additionally generated *RETREG1*-, *RETREG3*-, and *TEX264*-knockout cell lines, which were validated by genomic sequencing and Western blotting as shown in Fig. S1–S4.

**Reviewer #3:** In this manuscript, the authors explore the mechanism by which the MLV glycoGag protein counteracts the antiviral effect of SERINC5. The authors show that glycoGag utilizes selective autophagy to reduce the cellular levels of SERINC5. The authors show that glycoGag is highly unstable with a very short half life and localizes in internal organelles (presumably). They provide evidence that Nef and glycoGag utilize different mechanisms to downregulate cellular levels of SERINC5 in the cell. In addition, the authors show that the glycoGag effect on SERINC5 is dependent on the host protein RETREG1, an ER-phagy receptor, and is mediated by micro-ER-phagy.

However, a number of important weaknesses have been identified. The fact that the vast majority of the findings have been performed in the context of co-transfections in HEK293T cells and using the human ortholog of SERINC5 significantly weaken the relevance to natural infection of MLV. In fact, processes like autophagy are often regulated by multiple viral proteins during infection, thus by transfecting only glycoGag the authors may not be seeing what happens under physiological conditions during MLV infection.

We respectfully disagree with the suggestion to repeat nearly all experiments in murine systems. Validating such a comprehensive dataset, including the regeneration of over 10 knockout cell lines in murine cells, would be highly resource-intensive and lacks sufficient scientific justification.

The Nef-like activity of glycoGag in enhancing viral replication was indeed identified in human cells by Pizzato (PNAS, 2010, PMID: 20439730) and validated by Gottlinger (JVI, 2014, PMID: 24403584). Moreover, the glycoGag-mediated antagonism of SERINC5, as reported by the Pizzato and Gottlinger labs, was also established using human cells such as HEK293T cells (Nature, 2015, PMID: 26416733; Nature, 2015, PMID: 2015, PMID: 26416734). Other research groups (Rein, mBio, 2016, PMID: 27879338; Compton, mBio, 2020, PMID: 31964738) as well as our studies (PMID: 30862674, 30355687) have similarly utilized HEK293T and HeLa cells to investigate this mechanism. Thus far, no differences have been observed between murine and human cells in this context, making the study of glycoGag antagonism in human cells standard practice in the field.

Additionally, ubiquitin E3 ligase and autophagy genes are highly conserved across species, and we do not see compelling reasons to believe they would function differently in humans and mice.

Nonetheless, we acknowledge that testing in murine cells with the murine SERINC5 ortholog would provide valuable confirmation. We already performed MLV infection in NIH3T3 cells and confirmed glycoGag instability (Fig. 2A). Furthermore, we investigated both murine SERINC5 and its human ortholog in terms of trafficking and antiviral activity using authentic MLV, obtaining identical results (Fig. 4B, 4C, 4D, 4E).

To further address the reviewer's concerns, we have added the following new results:

1. We show that glycoGag binds to murine SERINC5 and its K131R mutant, similar to its binding with human SERINC5 (Fig.4G).
2. We demonstrate that glycoGag requires RETREG1 to downregulate murine SERINC5, as it does with human SERINC5 (Fig.7D).
3. We show that glycoGag facilitates the interaction between murine SERINC5 and RETREG1, as observed with human SERINC5 (Fig.7E).

These new findings further support the conclusion that glycoGag does not encounter a species-specific barrier between humans and mice.

Moreover, most experiments are performed at least twice or no information is provided about the number of replicates affecting the rigor of this study. Finally, the paper needs to be better edited with missing information (e.g., Fig 5D legend) and not enough information is provided in the material and methods section (e.g., transfection conditions, antibody concentrations etc).

The number of replicates is provided. Fig.5D legend has been corrected. Transfection conditions and antibody concentrations are also provided in Materials and Methods.

In line 665, Figure 1A legend, MLV Gag is not translated from an alternatively spliced RNA but from a full length genomic RNA as is the case for all retroviruses. Please fix this.

This has been corrected.

In line 122, the authors state that the half-life of glycoGag, based on Figure 2B data, is 15 minutes. This is incorrectly stated. The authors in Figure 2B are using 0 and 2 hour time points and they lose signal at some point between these two time points. It is not clear how they draw the conclusion of 15 minutes from the data presented in 2B. This statement needs to be rewritten to accurately reflect the data shown.

The half-life of glycoGag was estimated to be approximately 15 minutes based on Fig. 2A, instead of Fig.2B. To be more accurate, we have revised this to “less than 30 min”.

For Figure 2C, the experiment is not described in the materials and methods, which made it difficult to follow at times. Furthermore, if they see no effect with 3MA, Ly and Baf, how do they know their treatment worked? Controls for this experiment are missing.

The experimental protocol has been updated in the Materials and Methods section. Their effectiveness has been confirmed from Fig.5A and Fig.8B.

Figure 2D, the Cul3-KLHL20 E3 ligase is needed for glycoGag/glycoMA degradation in proteasomes in 293T cells. These viral proteins are found in MLV and as such are present in mouse cells in infections under physiological conditions. So, is this E3 ligase needed the same for proteasomal degradation of glycoGag/glycoMA under physiological conditions? The authors need to test this in mouse cells as they are more physiologically relevant than human cells and upon infection.

Human and mouse Cullin-3 share nearly complete sequence homology, supporting the cross-species relevance of our findings. Furthermore, the function of the Cul3-KLHL20 E3 ligase complex is highly conserved, and we have no evidence suggesting species-specific differences in glycoGag/glycoMA degradation. While we agree that testing in murine cells would provide additional confirmation, the current data in human cells offer a valid and informative model. Nonetheless, we recognize this as a valuable future direction to further strengthen the physiological relevance of our findings.

In Figure 3A, it would be better if localization of glycoGag/glycoMA was performed in murine cells. Also, the authors state that Nef is found in the plasma membrane but glycoGag/glycoMA are in the cytosol (lines 137-138). The authors should use markers to verify that Nef is on the plasma membrane in their system and it would be more informative if they defined where in the cytoplasm is glycoGag/glycoMA.

We believe there is no compelling reason to expect that glycoGag/glycoMA would localize differently in murine versus human cells, given their conserved function and the lack of reported differences in prior studies.

As for Nef, its plasma membrane localization in our system is supported by our previous findings showing its enrichment in lipid rafts (PMID: 11516650). As suggested by this reviewer, we expressed Nef-GFP in HeLa cells and stained the cells with the plasma membrane marker (DiIC18). Nef is clearly localized to the plasma membrane.

In addition, we used ER marker in Fig.5C and showed that glycoGag colocalized with the ER marker CALR, confirming its cytoplasmic localization.

At present, the precise cytoplasmic localization of glycoGag/glycoMA remains unclear, and identifying specific markers to define this is an important direction for future work.

In Figure 3B, it is not clear why SER5 and glycoMA are in the cytosol fractions. The kit (Beyotime P0033-1) they are using is purifying total membranes in the cell (mitochondrial, Golgi, PM and ER) vs. cytosolic fraction which contains all proteins found in the cytosol (non-membrane bound proteins). How is glycoMA and SER5 in the cytosolic fraction when they have transmembrane domains? glycoMA and Ser5 should only be found in the membrane fraction.

We have clarified that the Beyotime kit selectively purifies the plasma membrane, based on our investigation of SERINC5 membrane trafficking (*Nat Commun*, 2022; PMID: 35474067).

We speculate that during secretion, glycoGag and SERINC5 may be enclosed within transport vesicles—such as those derived from the ER or Golgi—and may transiently appear in the cytosol during active trafficking.

In Figure 3B, which band is Nef and which one is glycoMA in the middle blot? The only bands shown are running at the same height, which is confusing as Nef and glycoMA are not of the same size (as shown in Fig. 3C. This blot is confusing.

The Nef and glycoMA bands are now specified in Fig. 3C.

In Fig3C, it would be more physiologically relevant if this coIP was performed with mouse SERINC5 and not the human ortholog.

As suggested, we conducted this experiment and confirmed that glycoGag also binds to murine SERINC5. These new results are presented in Fig.4G.

In Figure 4B, the authors must explore the localization of mouse and human SER5 using markers for the different organelles with both wildtype and K130 or 131R. The authors stating in line 160-161 that wildtype SER5 is found on the plasma membrane is incorrect. This conclusion can only be drawn if they perform co-staining with a PM marker.

We previously demonstrated that wild-type human SERINC5 localizes to the plasma membrane, whereas the K130R mutant is retained in the cytoplasm, as shown by co-staining with a plasma membrane marker (*Nat Commun*, 2022; PMID: 35474067).

Here, we confirm a similar localization pattern for murine SERINC5 and its K131R mutant by comparison with their human counterparts (Fig.3B). Results are further confirmed by biochemical purification (Fig.3C).

The majority of experiments in figure 4 that demonstrate differences between Nef and glycoMA on SER5 are performed in the context of transfections with either 293T cells or HeLas. Nothing is done in murine cells or in the context of infection. Moreover, panels 4F-J are performed using human SERINC5. Experiments using glycoMA should be performed with murine SER5 and not the human orthologs.

As suggested by the reviewer, we have added new data showing that glycoGag also binds to murine SER5 (Fig.4G). However, we see no compelling evidence to suggest that glycoMA and SERINC5 traffic or function differently in human versus murine cells, as previously discussed.

In figure 4F, control blots showing absence of KLHL20 and Cul3 are not provided and should be included in this figure panel.

These knockout reagents have already been validated in our previous publication (*Nat Commun*, 2022, PMID: 35474067).

Figure 4I and J, the requirement for CCNK and CDK3 with glycoMA is not absolute. While there is no complete rescue of SER5 in the case of glycoMA, when CCNK and CDK13 are knocked down, there is still more protein present in lanes 8 when compared to lanes 6. So the authors cannot state that CDK13 is not required for downregulation of SER5 (line 190). These findings suggest that there might be an additional factor involved along with CCNK and CDK13. This needs to be rewritten.

As suggested, this statement has been rewritten.

The authors state in lines 191-192 that the data “confirm that SER5 downregulation by GlycoGag does not necessarily require Ser5 on cell surface”. This is an overinterpretation of the data. The findings suggest that, but only if the authors block SER5 translocation to the PM, and still see reduction in SER5 levels, they can make that statement.

We blocked the human and murine SER5 trafficking to the PM by introducing the K130R and K131R mutation and found that glycoGag reduces SER5 levels (Fig.4E), which supports our statement.

For Figure 5B, the authors are performing experiments on SER5 localization in HeLa cells with either Nef or GlycoMA. This is not the appropriate cell type or SER5 ortholog used. The authors ought to use a murine cell line and use mouse SER5 to say anything about the effect of GlycoMA on SER5 localization. Moreover, this experiment needs to be performed in the presence of MLV infection.

As noted earlier, investigating glycoGag antagonism in human cells is a widely accepted and established approach. To date, no functional differences have been observed between murine and human cells in this context. Furthermore, using MLV infection would preclude the inclusion of Nef as a critical control, which is essential for our comparative analysis.

For Figure 5B and C, the authors need to employ additional quantitative approaches on their imaging to confirm LAMP1 or CALR colocalization.

As suggested by this reviewer, we added Person’s Correlation Coefficient (PCC) to these figures, which enhances the quality and clarity of the data presentation.

Figure 5D legend is missing. In fact it says “Write down method:…”

It has been corrected.

There is a figure legend 5E, but there is no 5E figure.

It has been corrected.

For Figure 6, it would be beneficial for the authors to quantify band intensity. Also there is no mention in the figure legend about number of replicates for this experiment.

As suggested by this reviewer, quantification of the RETREG1 activity is now provided in Fig. 6C. The number of replicates is also provided.

In Figure 6, there are panels that are missing their labeling. It is assumed that it is the ER-phagy receptors, but it would be easier to the reader to provide that next to the blot.

The labeling is now provided.

All data on Figure 7 are in HEK293T cells, which are not physiologically relevant to glycoGag and in extension to MLV. The authors need to do these experiments in murine cells using mouse SER5. Also, RETREG1 used in this assays is the human ortholog. It is never shown what happens with the murine ortholog and most importantly during infection in murine cells.

Generating and validating the full set of these knockout lines in murine cells would be highly resource-intensive. Existing evidence does not suggest functional differences between human and murine RETREG1 in this context, and we believe the mechanistic insights gained from human cells remain informative and valid.

Figure 7A and C, there should be quantification of the SER5 bands in the different conditions, as that would be more convincing.

As suggested by this reviewer, these quantifications are now provided.

Looking at the SER5 western blot panels in Figure 7C, it shows that K130R SER5 is unaffected by the presence of glycoGag in lane 2. This is confusing, as all the data from prior figures (e.g., fig 7A and B) clearly show a reduction of K130R SER5 in the presence of glycoGag. The authors need to address that, as it contradicts their previous findings.

This experiment was done in *RETREG1*-knockout cells, which is consistent with previous results.

Figure 8, quantification of the SER5 bands showing the reduction of SER5 under the different conditions/knockout cells will add rigor to the data shown.

Quantification of SER5 is now provided in Fig.8B. The glycoGag-mediated downregulation of SER5 in Fig. 8A (lanes 27–72) is visually apparent; therefore, additional quantification was not included due to space constraints.

Furthermore, Figure 8 would be better done in the context of MLV infection in murine cells, as it would show the effect of glycoGag on SER5 during microER-phagy under physiological conditions.

As noted previously, generating and validating the complete set of these knockout lines in murine cells would require substantial resources and lack scientific justification. We believe the current model already provides meaningful mechanistic insights.
