# Supplementary material for: Murine leukemia virus glycoGag antagonizes SERINC5 via ER-phagy receptor RETREG1
Source: PLoS Pathog. 2025 Oct 9;21(10):e1013023. doi: 10.1371/journal.ppat.1013023 (PMC12530543; doi:10.1371/journal.ppat.1013023)
Supplement: Attachment — Submitted filename: Point-by-point letter.docx [file ppat.1013023.s009.docx]

Reviewers' Comments:

Part I - Summary

**Reviewer #4:**

The authors reported a new mechanism (via ER-phagy) for GlycoGag to down regulate serinc5. The experiments are well designed and the results support authors' major conclusion. The report should excite people who study host-virus interactions.

This reviewer does have some concerns.

We thank this reviewer for these encouraging comments. The concerns have been addressed.

**Reviewer #5:**

Overall, the paper is rich with data and the findings are interesting. They appear to have significance for the mechanism of Ser5 downregulation by the MLV glycoGag protein. The impact may be limited by the observation that RETREG1 appears at least partially dispensable for downregulation of wild type Ser5. Perhaps it would be informative to perform assays of glycoMA rescue of HIV-1 infectivity from Ser5 inhibition in a series of cotransfection assays in RETREG1-deficient vs. control cells.

We also thank this reviewer for these encouraging comments. As suggested, we have performed assays of glycoMA rescue of HIV-1 infectivity from Ser5 inhibition in RETREG1-KO cells (see our response to Reviewer #4 comment 1). These new results are shown in **Fig. 8B.**

Part II – Major Issues: Key Experiments Required for Acceptance

**Reviewer #4:**

1. It seems glycoGag directs non-cell-surface (ER) serinc5 for degradation via ER-phagy and directs cell-surface serinc5 “likely …. in endolysosomes”. Because it is the cell-surface serinc5 that inhibits virus replication, the authors should examine the real contributions of ER-phagy in serinc5 antagonism by glycoGag. The authors can perform the virus infectivity assay (as in Figure 1B) in RETREG1 KO cells to test to which extent the KO could reduce glycoGag’s antagonism activity.

We thank this thoughtful comment. As suggested, we conducted this experiment, and the new results are shown in **Fig. 8B**. RETREG1-KO greatly reduced glygoGag’s antagonism activity to Ser5, but it did not the Nef’s antagonism activity. These results confirm the real contributions of ER-phagy in Se5 antagonism by glycoGag.

2. All experiments were performed using over-expressed glycoGag. I agree with reviewer#3 that it is important to test glycoGag’s function at physiological level. The authors can infect WT and RETREG1 KO NIH3T3 cells with MLV (as in Figure 2A) and then examine the degradation of WT and K130R serinc5.

We thank this reviewer again for this suggestion. As suggested, we expressed murine Ser5 (mSer5) and its K131R mutant in NIH3T3 cells in the presence of siRNAs to *RETREG1* and infected these cells with MLV. We found that MLV infection effectively downregulated mSer5 but not the K131R mutant, and RETREG1-knockdown blocked the downregulation. These new results are shown **Fig. 8A**, which confirms the glycoGag activity at physiological levels.

**Reviewer #5:**

1. Fig. 1A. Authors have concluded that glyco-Gag adopts a (unexpected) topology in the membrane based on amino acid sequence alone (line 103). This could be correct, but in the absence of confirmation (e.g. by staining of cells with an MLV CA antibody), seems speculative.

As we introduced in the text (line 97), the Gottlinger lab initially reported glycoGag as a type II transmembrane protein (Ref #24). They showed that glycoGag has a cytoplasmic domain containing an AP2-sorting motif, which we confirmed later (Ref #9).

As suggested by this reviewer, we also stained glycoGag with an anti-p30 antibody and detected a similar intracellular distribution of glycoGag as shown in Fig. 3A.

2. Fig. 4I. Authors claim that glycoMA can downregulate Ser5 under conditions in which Nef cannot. In these experiments, the ratios of levels of glycoMA vs. Nef seem much greater than those in other experiments (e.g. 4E). In these experiments, were cells transfected with the same amounts of glycoMA and Nef expression plasmids? If so, then why do the levels appear to be so different?

Yes, the same amounts of glycoMA and Nef expression plasmids were used in these experiments. In Fig. 4E, both Nef and glycoMA were detected on the same Western blot membrane, allowing for direct comparison. In contrast, Fig. 4I shows them on separate blots. We have previously demonstrated that glycoGag is significantly less stable than Nef due to rapid proteasomal turnover (see Fig. 2). Additionally, in Fig. 4I, the glycoMA blot was exposed for a longer duration than the Nef blot during detection, which likely contributed to the apparent differences in expression levels.

3. Fig. 4J. Authors draw conclusions regarding downregulation of Ser5 mutants from images of one or two cells. This falls short of establishing reproducibility.

We acknowledge the concern and would like to clarify that the confocal imaging data shown in Fig. 4J are supported by two independent sets of Western blot results (Fig. 4H and Fig. 4I), which already demonstrated the differential ability of Nef and glycoGag to downregulate the S360A mutant. To further strengthen the evidence and address the issue of reproducibility, we have replaced images containing one cell with those containing multiple cells.

Also, Ser5.S360A levels appear to vastly exceed those of WT and S249A in Fig. 4H. Is the apparently lack of downregulation simply owing to excessively high levels of the S360A mutant?

We acknowledge that Ser5 S360A was expressed at higher levels than the WT and S249A variant in Fig. 4H. However, our previous work (Ref #20) has demonstrated that even when S360A is expressed at lower levels than WT or S249A, Nef still fails to downregulate it—while it effectively downregulates WT and S249A. In contrast, glycoMA remains capable of downregulating S360A even when its expression is higher. This further supports the conclusion that Nef and glycoGag utilize distinct mechanisms to downregulate Ser5.

4. Fig. 5D: It would be helpful to confirm the key associations detected by MS by IP-western.

We have confirmed that Ser5 and glycoGag can pull down RETREG1 by IP-western (**Fig. 7E**).

Part III – Minor Issues: Editorial and Data Presentation Modifications

**Reviewer #4:**

1. The expression of serinc5 K130R in RETREG1 KO cells is not consistent in Figure 7A and Figure 7D. In 7A, serinc5 is expressed at obviously lower level in KO cells, but in 7D, the expression levels in WT and KO cells are comparable. Is this due to different transfection efficiency in different experiments?

Yes, the observed difference in Serinc5 K130R expression levels between Fig. 7A and Fig. 7D is likely due to variations in transfection efficiency across independent experiments.

2. Figure 3C. The authors concluded that “glycoGag is targeted to proteasomes for degradation”. However, in Figure 5A, proteasome inhibitor MG132 treatment did not increase the levels of glycoGag.

We have repeated the experiment shown in **Fig. 5A**, and the updated results demonstrate that glycoGag levels do increase upon MG132 treatment. The figure has been revised accordingly to reflect this finding.

3. Figure 5 showed that glycoGag strongly localized in ER. Then why glycoGag cannot be detected in the membrane fraction (Figure 3B).

The membrane fractionation method used in our study isolates only the plasma membrane, as described in our previous report (Ref #13). Therefore, glycoGag localized in other compartments such as the ER would not be detected using this approach.

4. The authors stated that “serinc5 significantly increased the levels of glycoMA in the membrane fraction” (line 145). I am wondering why only serinc5, but not glycoGag, is degraded in the membrane fraction.

While glycoGag steady-state levels appear elevated, this does not necessarily indicate an absence of membrane-associated degradation. Therefore, we would not conclude that glycoGag is not subject to degradation in the membrane fraction.

5. Line 240, “K130” should be “K130R”.

It has been corrected.

**Reviewer #5:**

1. The abstract could be improved by rewriting for a broader audience. I would remove the phrases “employing the K130R mutant” (line 27); “while still in the cytoplasm” (line 30). I also found the sentence in lines 30-32 to be confusion: are the authors claiming that SERINC5 binds glycoGag in the cytoplasm and moves it to the plasma membrane, only to be downregulated by it? This seems paradoxical. I would also de-emphasize the method of MS in the abstract, as this is an unnecessary detail. Finally, the authors end both the Abstract and Discussion with a tag line about the arms race between retroviruses and Ser5. What exactly are these “critical new insights?” Aren't they just mechanistic details of Ser5 downregulation by different viral proteins?

We sincerely appreciate these thoughtful comments. In response, we have thoroughly revised the Abstract to improve clarity and accessibility for a broader audience. Specifically, we have removed the phrases “employing the K130R mutant” and “while still in the cytoplasm,” clarified the wording around SERINC5’s interaction with glycoGag to avoid ambiguity, and de-emphasized mass spectrometry as a methodological detail. Additionally, we have removed the concluding tag line from both the Abstract and the Discussion to avoid redundancy and better reflect the mechanistic focus of our findings.

2. Fig. 1C. Authors directly compare levels of Nef and glycoMA based on band intensities on an immunoblot. Presumably, both bands were detected by an HA-specific antibody, but this was not stated.

Yes, this is now stated in the Fig. 1C legend.

3. Line 200: change to: “…require Ser5 accumulation at the cell surface.”

This has been changed as suggested.

4. Line 214: change to: “…but also showed some intracellular colocalization with CALR.”

This has been changed as suggested.

5. Lines 285-286: Authors should comment on the apparent stabilization of glycoMA by 3-MA. What does this suggest regarding PI3KC3 and/or autophagy and glycoMA stability?

As shown in Fig. 9A, PIK3C3 is not required for glycoGag-mediated downregulation of Ser5 via autophagy. Therefore, we speculate that 3-MA may inhibit this process by targeting a different, unidentified PI3K. Since 3-MA blocks Ser5 downregulation, Ser5 may, in turn, stabilize glycoGag—a relationship we previously demonstrated in Fig. 3D.
